# Prospective Evaluation of the Impact of Sedation During LAAO on Cognitive Function Using Oculomotor Biomarkers

**DOI:** 10.1016/j.jacadv.2025.102090

**Published:** 2025-08-22

**Authors:** Agata Sularz, Alejandra Chavez-Ponce, Ghasaq Saleh, Gurpreet Singh, Bilal Unar, Trevor Simard, Matias Shulz, Gerardo Fernandez, Danilo Verge, Mohamad Alkhouli

**Affiliations:** a Department of Cardiovascular Medicine, Mayo Clinic, Rochester, Minnesota, USA; b Department of Cardiovascular Medicine, Mayo Clinic Health System, Eau Claire, Wisconsin, USA; c Viewmind Inc., New York, New York, USA

**Keywords:** anesthesia, cognitive assessment, left atrial appendage occlusion, postoperative delirium

## Abstract

**Background:**

Intracardiac echocardiography guidance has emerged as a feasible alternative to transesophageal echocardiography in guiding left atrial appendage occlusion (LAAO). A proposed advantage of this approach is the avoidance of cognitive impairment associated with general anesthesia (GA) in elderly patients.

**Objectives:**

We studied the effect of anesthesia method (GA vs moderate sedation [MS]) on cognitive function using a novel digital oculomotor biomarker.

**Methods:**

Patients undergoing LAAO were prospectively enrolled at 2 medical centers (GA center and MS center). Cognitive function was assessed at baseline (V1), 6 hours post-LAAO (V2), and at 45 days (V3) using the ViewMind system, which consists of a head-mounted device with eye-tracking capabilities. We examined differences in cognitive function as measured by 2 oculomotor tests (Go/No Go and N-Back), within each group between V1 vs V2 and V1 vs V3.

**Results:**

Forty patients (20 in each group: GA and MS) were enrolled. Patients in the MS group showed significant long-term (V1 vs V3) improvement in working memory (Δ *n* complete sequences, *P* = 0.005), processing speed (Δ *n* wrong fixations, *P* = 0.050), and sustained attention (gazing recognition (ms), *P* = 0.010), as measured by the N-Back task while there were no differences in the GA group (*P* = 0.180 for Δ *n* complete sequences, *P* = 0.410 for Δ *n* wrong fixations on hitbox, and *P* = 0.310 gazing recognition [msec]).

**Conclusions:**

Memory-based oculomotor metrics improved after the procedure in patients undergoing LAAO under MS but not within the GA group. This proof-of-concept study supports a differential impact of sedation strategies on cognitive function in elderly patients undergoing LAAO.

Left atrial appendage occlusion (LAAO) is a feasible alternative to oral anticoagulation in high bleeding risk patients with nonvalvular atrial fibrillation.^1^ In the United States, over 96% of LAAOs are performed under general anesthesia (GA).^2^ However, several studies have documented that LAAO can be performed safely and effectively with moderate or even minimal sedation.^3^, ^4^, ^5^ Use of general anesthetics has been associated with subclinical cognitive impairment and postoperative delirium after surgical interventions.^6^^,^^7^ Conversely, the use of moderate sedation (MS) with transcatheter procedures has been associated with lower mortality, shorter hospital stays, and more frequent home discharges.^8^ Given that most LAAO patients are elderly, frail, and burdened with comorbidities, reducing anesthetic burden in this population is a priority.^9^

Oculomotor behavior encompasses various eye movements, including saccades (rapid shifts in gaze), smooth pursuit (tracking moving objects), and fixation (sustaining gaze on a stationary target), among others.^10^ These movements are tightly linked to cognitive function.^11^^,^^12^ Hence, disturbances in oculomotor behavior have been proposed as a potential biomarker of cognitive impairment in patients with neurodegenerative conditions.^13^, ^14^, ^15^ The aim of this multicenter study was to assess the impact of the type of sedation regimen on cognitive function using a novel digital oculomotor biomarker (ViewMind Atlas) in patients undergoing LAAO with the WATCHMAN FLX or AMULET device.

## Methods

### Patient recruitment

This study was a prospective, nonrandomized trial which enrolled patients undergoing LAAO at 2 institutions (Mayo Clinic, Rochester, and Mayo Clinic Health System in Eau Claire). Patients underwent the procedure under GA at Mayo Clinic Health System in Eau Claire or under MS in Mayo Clinic, Rochester. Peri-operative care was completed as per standard of care practices at each institution and was not affected by the study. We included patients ≥50 years of age with a clinical indication for LAAO. Participants provided written informed consent and were asked to return for the 45-day follow-up (±7 days). Patients with significant cognitive or visual impairment were excluded from the study. Patients who were not primarily English speakers were also excluded. The choice of LAAO device (Amulet vs WATCHMAN FLX) was left at the discretion of the operating physician. The study was approved by a local Institutional Review Board (22-002761). The LAAO procedure was not impacted by the cognitive testing and the operators were blinded to the results.

### Cognitive assessment and oculometric variables

**Figure 1:**
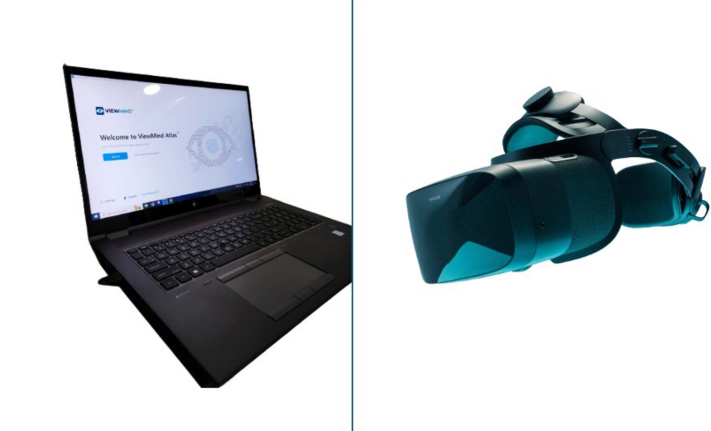
Computer and Head-Mounted Display Compatible With ViewMind Atlas Cognition App

**Figure 2:**
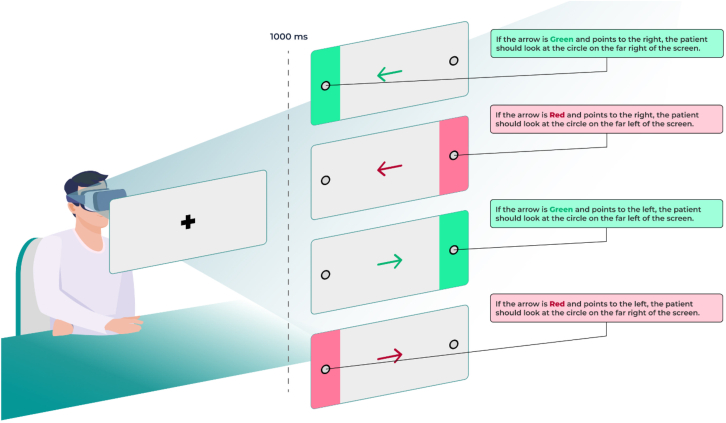
Go/No-Go Visual task Two examples are shown below. The circle highlighted in green on the screen indicates the direction of the gaze.

Cognitive function was assessed at baseline (before the procedure, V1), after the procedure but before discharge (>6 hours post-LAAO, [V2]), and at the follow-up visit on day 45 (V3). The ViewMind Atlas system, consisting of an head-mounted device with eye-tracking capabilities was used for all assessments (Figure 1). A trained evaluator assisted each patient in wearing the headset and provided verbal instructions. Following the initial calibration phase, the patient underwent 2 standardized ocular tracking tests: the Go/No-Go and the N-Back tasks. The oculometric responses of the patient to those tasks reflect working memory and executive function and were selected based on previous studies on postoperative cognitive impairment.^16^, ^17^, ^18^ We utilized the Go/No Go eye-tracking task to evaluate cognitive performance across prespecified domains, including processing speed and executive function^19^^,^^20^ (Table 1). Participants were asked to shift their gaze in response to arrows that appeared at the center of the screen. The arrows varied randomly in direction (right or left) and color (green or red) during each trial (Figure 2). Participants were instructed to look toward the fixation point indicated by the arrow when the color was green, or away from it, when the color was red. Here, we measured reaction times (exam duration) and correct responses (% of correct fixations). This task consisted of ∼50 trials.

**Table 1:** Cognitive Domains Studied as Stratified by the Cognitive Task and Oculometric Variables

Oculometric	Cognitive Domain	Brain Region
Go/No Go Task		
Correct fixations (%)	Executive function (Planning)	Frontal or subcortical
Exam duration (min)	Processing speed	Frontal
N-Back task		
Completed sequences (n)	Working memory	Frontal or temporal
Start fixations off-center (n)	Executive function (Planning)	Frontal
Wrong fixations on hitbox (n)	Executive function	Frontal or subcortical
Gazing recognition (msec)	Sustained attention	Frontal or parietal

**Figure 3:**
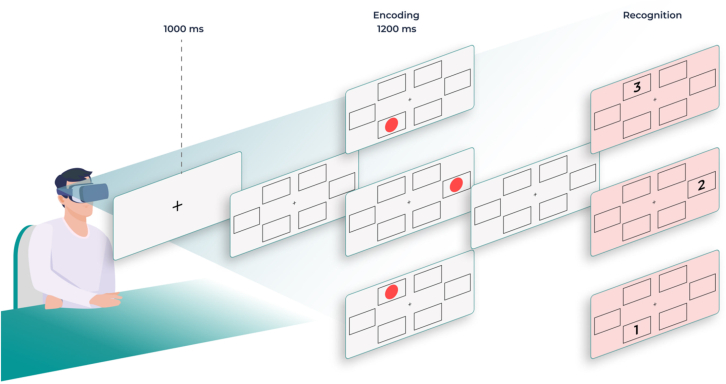
N-Back Visual task During the task, red circles appear sequentially within the peripheral rectangles, while the patient fixates his or her gaze on the central cross. After the sequence, the patient must quickly look at the rectangles in the reverse order to the appearance of the circles.

**Figure 4:**
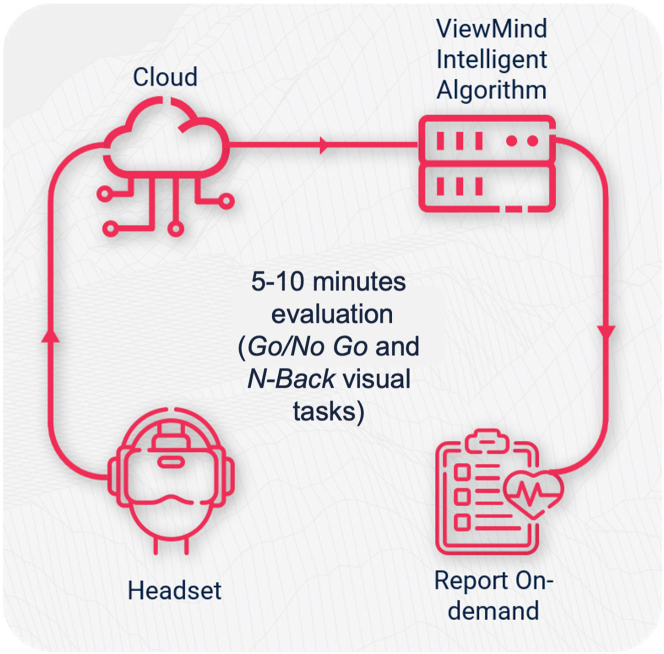
The Study Flow Chart

The N-Back task was designed to assess working memory, executive function and sustained attention. Each trial consisted of 2 stages: “encoding” and “recognition” (Figure 3). During the encoding stage, participants saw a sequence of 3 red circles appearing in “N” boxes. Throughout this stage, they were instructed to fix their gaze at a cross presented in the center of the screen and rely on their peripheral vision to memorize the location of the “N” boxes in which the circles appeared. In the “recognition” stage, participants were then asked to direct their gaze to the “N” boxes in reverse order. For example, if the encoding sequence was “N” box: 1-2-3 the participants were expected to retrace it in reverse, ie, 3-2-1. Here, we measured the number of complete sequences (the *n* of times participants correctly completed the full N-back sequence), gazing recognition speed (the speed of information processing measured as the time taken to correctly recognize the sequence of targets), and the number of wrong fixations (both away from the central fixation point and N boxes/hitbox). Additionally, during the recognition stage, participants had to start the activity from the center of the screen; if this was not done, this behavior would be understood as an alteration in executive function (start fixation off-center) (Table 1). The first 10 responses were used as practice and discarded. The trials were repeated ∼100 times. Figure 4 shows the study flow chart. Both tasks took ∼10 minutes to complete.

### Statistical analysis

Data on cognitive testing and key procedural variables were collected for the 2 groups (MS and GA). Continuous variables are expressed as mean ± SD or median (IQR), where appropriate, and discrete variables as counts and percentages. To assess the change in cognitive function within groups, we examined differences in ocular performance as measured by the 2 oculomotor tests (Go/No Go and N-Back), within each group between V1 vs V2 and V1 vs V3. Statistical analyses were performed using Statistics Package for the Social Science, version 28.0.0.0. A paired *t*-test or Wilcoxon signed rank test were used as appropriate to compare within-group performance changes (Δ) between V1 and V2 and between V1 and V3. Statistical significance was defined as *P* < 0.050 for all comparisons.

## Results

Forty patients (20 in each group: GA and MS) were enrolled. Table 2 summarizes baseline clinical and procedural characteristics of each group. Age, female sex, HAS-BLED, and CHA_2_DS_2_-VASc scores were similar between the 2 groups (ie, 79.0 vs 79.0 years; 14 vs 14%; 3.0 vs 3.5; 4.0 vs 5.0, respectively). Selected neurocardiovascular risk factors and comorbidities were also very similar between the 2 groups.

**Table 2:** Baseline Characteristics of the Study Groups (General Anesthesia vs Moderate Sedation)

	Moderate Sedation (n = 20)	General Anesthesia (n = 20)	*P* Value
Age, y, median (IQR)	79.0 (72.8-84.2)	79.0 (70.8-82.0)	0.533
Female, n (%)	14 (70.0%)	14 (70.0%)	1.000
BMI, Kg/m^2^, median, (IQR)	29.1 (26.5-33.9)	27.8 (24.1-35.6)	0.543
BSA, m^2^, median, (IQR)	2.0 (1.9-2.1)	2.0 (1.7-2.3)	0.745
HAS-BLED score, median (IQR)	3.0 (2.0-4.0)	3.0 (2.8-4.0)	0.517
CHA_2_DS_2_-VASc score, median, (IQR)	4.0 (3.8-5.0)	5.0 (3.8-6.0)	0.271
Smoking, n (%)	14 (70.0%)	12 (60.0%)	0.741
Alcohol use, n (%)	10 (50.0%)	9 (45.0%)	1.000
Hypertension, n (%)	16 (80.0%)	17 (85.0%)	1.000
Diabetes mellitus, n (%)	5 (25.0%)	6 (30.0%)	1.000
Dyslipidemia, n (%)	15 (75.0%)	16 (80.0%)	1.000
Coronary artery disease, n (%)	9 (45.0%)	9 (45.0%)	1.000
Prior myocardial infarction, n (%)	5 (25.0%)	8 (40.0%)	0.501
Prior TIMI major or multiple bleed, n (%)	4 (20.0%)	4 (20.0%)	1.000
Prior neurological bleed, n (%)	4 (20.0%)	4 (20.0%)	1.000
Atrial fibrillation and/or atrial flutter, n (%)	20 (100.0%)	20 (100.0%)	1.000
Transient ischemic attack, n (%)	3 (15.0%)	2 (10.0%)	1.000
Ischemic stroke, n (%)	3 (15.0%)	3 (15.0%)	1.000
LAAO device Size, mm, median (IQR)	31.0 (24.8-35.0)	27.0 (24.0-31.0)	0.126
Device type: Amplatzer Amulet, n (%)	4 (20.0%)	0 (0.0%)	0.107
Device type: Watchman FLX, n (%)	16 (80.0%)	20 (100.0%)	0.107
Intraprocedural medication use			
Propofol, n (%)	0 (0)	20 (100)	∝
Midazolam, n (%)	20 (100)	0 (0)	∝
Lidocaine, n (%)	20 (100)	20 (100)	∝
Rocuronium, n (%)	0 (0)	19 (95)	∝
Succinylcholine, n (%)	0 (0)	1 (5)	1.000
Fentanyl, n (%)	20 (100)	19 (95)	1.000

BMI = body mass index; LAAO = left atrial appendage occlusion.

**Figure 5:**
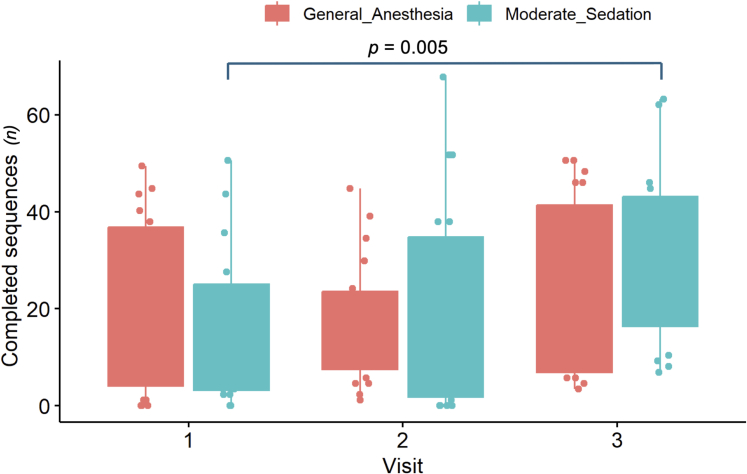
The Number of Complete Sequences During the N-Back Task A box-plot graph to demonstrate the performance of the general anesthesia and moderate sedation groups on the and N-back cognitive task as measured by the number of complete sequences.

**Figure 6:**
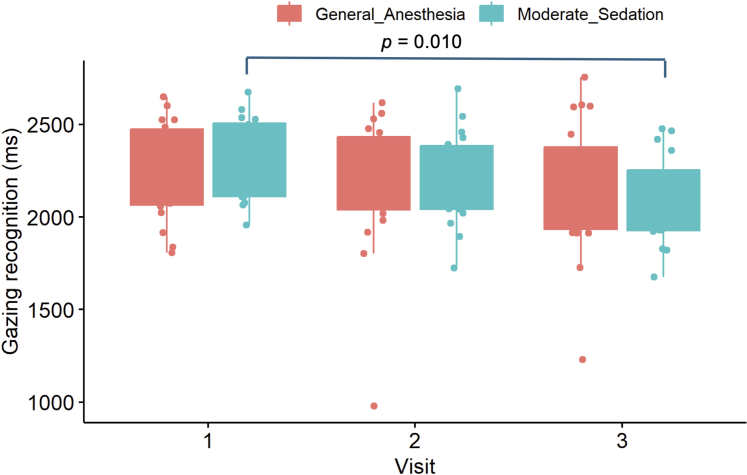
Gazing Recognition Speed (ms) During the N-Back Task A box-plot graph to demonstrate the performance of the general anesthesia and moderate sedation groups on the and N-back cognitive task as measured by gazing recognition speed (ms).

Table 3 shows the results of the within-group comparisons for each oculomotor test, stratified by the selected oculomotor task. In the N-Back task, the MS group showed significant long-term improvement in working memory, as measured by an increase in the *n* completed sequences between V1 vs V3 (8 [3.4-33.6] vs 29 [9.5-42.2], *P* = 0.005). There was no statistically significant long-term improvement in the performance of the GA group (20 [4.0-36.8] vs 17 [6.9-41.4], *P* = 0.180). Although not statistically significant, in the short-term (V1 vs V2), the GA group’s performance deteriorated (20 [4.0-36.8] vs 12 [7.5-23.6], *P* = 0.090), whereas the MS group performance improved slightly (8 [3.4-33.6] vs 17 [1.7-34.8], *P* = 0.580) (Figure 5). The MS group also showed a significant long-term improvement in processing speed, as measured by a decrease in gazing recognition (msec) (V1 vs V3 = 2,295 [2,084-2,483] vs2,068 [1,933-2,378] ms, *P* = 0.010). Although not statistically significant, GA group showed a trend towards improvement in the long-term (V1 vs V3 = 2,223 [2,066-2,474] vs 2,138 [1,929-2,323] ms, *P* = 0.310). There were no statistically significant differences in either group’s performance in the short-term, but the GA group’s showed deterioration in learning (V1 vs V2 = 2,223 [2,066-2,474] vs 2,374 [2,040-2,432] msec, *P* = 0.310) (Figure 6).

**Table 3:** Results of the ViewMind Atlas Oculometric Analysis by Sedation Group (General Anesthesia or Moderate Sedation), Visual Task (Go/No Go, N-Back) and Visit Timing

Visual Task (Variable, Unit)	Visit 1		Visit 2		*P* Value	Visit 3		*P* Value
	GA	MS	GA	MS		GA	MS	
Go/No Go (exam duration, min)	1.4 (1.3-1.8)	1.6 (1.4-1.8)	1.5 (1.4-1.7)	1.7 (1.4-2.0)	GA vs GA, *P* = 0.510 MS vs MS, *P* = 0.600	1.4 (1.3-1.5)	1.4 (1.3-1.6)	**GA vs GA, *P* = 0.001** MS vs MS, *P* = 0.10
Go/No Go (correct fixations, %)	88 (80.8-91.3)	86 (82.3-90.0)	87 (84.5-92.7)	86 (82.1-89.9)	GA vs GA, *P* = 0.920 MS vs MS, *P* = 0.650	89 (84.3-92.9)	87 (81.0-90.6)	GA vs GA, *P* = 0.080 MS vs MS, *P* = 0.780
N-Back (complete sequences, n)	20 (4.0-36.8)	8 (3.4-33.6)	12 (7.5-23.6)	17 (1.7-34.8)	GA vs GA, *P* = 0.090 MS vs MS, *P* = 0.580	17 (6.9-41.4)	29 (9.5-42.2)	GA vs GA, *P* = 0.180 **MS vs MS, *P* = 0.005**
N-Back (start fixation off-center, n)	32 (20-70)	17 (10-50)	24 (10-60)	9 (0-20)	GA vs GA, *P* = 0.930 MS vs MS, *P* = 0.270	33 (10-60)	5 (0-30)	GA vs GA, *P* = 0.160 MS vs MS, *P* = 0.090
N-Back (wrong fixation on hitbox, n)	2.2 (2.0-3.0)	1.7 (1.4-2.2)	2 (1.5-2.6)	1.6 (1.3-2.1)	**GA vs GA, *P* = 0.030** MS vs MS, *P* = 0.890	2.1 (1.5-2.9)	1.3 (1.0-2.0)	GA vs GA, *P* = 0.410 **MS vs MS, *P* = 0.05**0
N-Back (gazing recognition, msec)	2,223 (2,066-2,474)	2,295 (2,084-2,483)	2,374 (2,040-2,432)	2,196 (2,041-2,385)	GA vs GA, *P* = 0.310 MS vs MS, *P* = 0.190	2,138 (1,929-2,323)	2,068 (1,933-2,378)	GA vs GA, *P* = 0.310 **MS vs MS, *P* = 0.010**

Cognitive function was assessed at baseline (before the procedure, V1), after the procedure but before discharge (>6 h postleft atrial appendage occlusion, V2), and at the follow-up visit on day 45 (V3), shown as median (IQR). Significant *P* values are bolded.

GA = general anesthesia; MS = moderate sedation.

**Figure 7:**
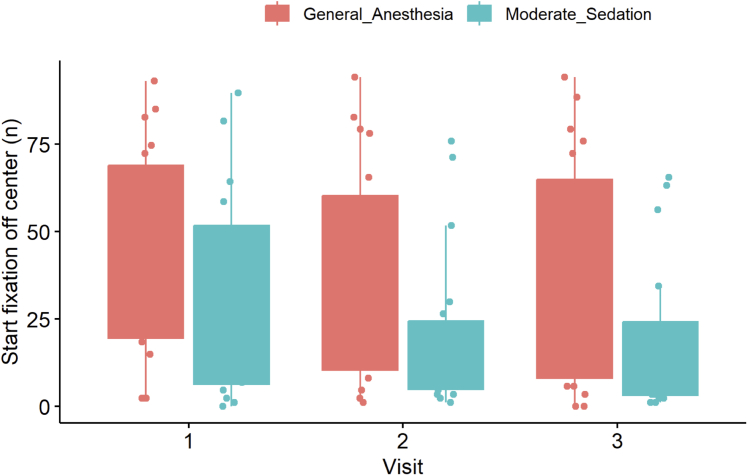
The Number of Start Fixations Off-Center During the N-Back Task A boxplot to demonstrate the performance of the general anesthesia and moderate sedation groups on the and N-back cognitive task as measured by the number of start fixations off-center (number).

**Figure 8:**
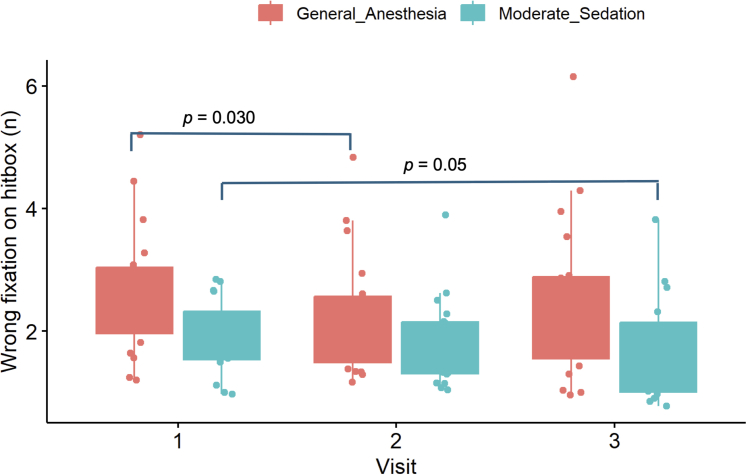
The Number of Wrong Fixations on Hitbox During the N-Back Task A box-plot graph to demonstrate the performance of the general anesthesia and moderate sedation groups on the and N-back cognitive task as measured by the number of wrong fixations on hitbox (number).

Executive function on the N-back task was assessed by 2 variables: the number of start fixations off-center and the number of wrong fixations on hitbox. For the *n* start fixations off-center, there were no statistically significant differences in performance between groups (both short term [V1 vs V2] and long-term [V1 vs V3]). However, numerically MS group made fewer errors long-term term (V1 vs V3: 17 [10-50] vs 5 [0-30], *P* = 0.090), whereas GA group made errors more frequently (V1 vs V3: 32 [20-70] vs33 [10-60], *P* = 0.160) (Figure 7). With regards to the *n* of wrong fixations on hitbox, there was a significant reduction in error rate in the MS group long-term (V1 vs V3: 1.7 [1.4-2.2] vs 1.3 [1.0-2.0], *P* = 0.050). GA group showed no long-term improvement (V1 vs V3: 2.2 [2.0-3.0] vs 2.1 [1.5-2.9], *P* = 0.410) but did significantly improve performance short-term (V1 vs V2: 2.2 [2.0-3.0] vs 2 [1.5-2.6], *P* = 0.030) (Figure 8).

**Figure 9:**
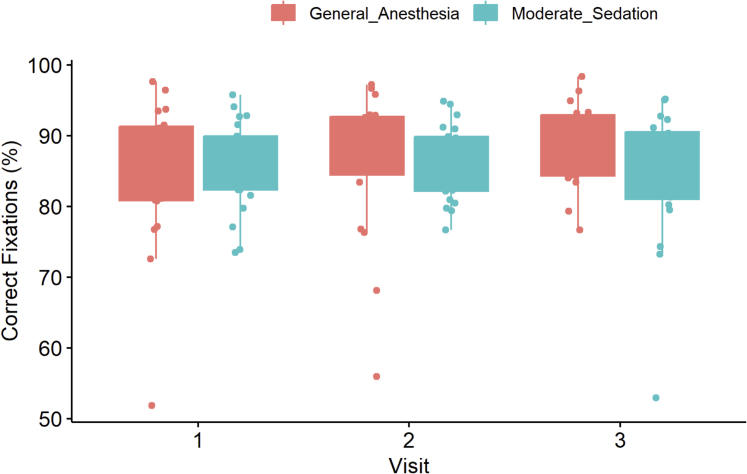
The % of Correct Fixations (%) During the Go/No Go Task A box-plot graph panel to demonstrate the performance of the general anesthesia and moderate sedation groups on the Go/No Go task as measured by the % of correct fixations (%).

For the % correct fixations in the Go/No Go task, there was a mild, nonsignificant long-term improvement in both the GA (V1 vs V3: 88 [80.8-91.3] vs 89 [84.3-92.9], *P* = 0.080) and MS (V1 vs V3 = 86 [82.3-90.0] vs 87 [81.0-90.6]) groups (Figure 9).

**Figure 10:**
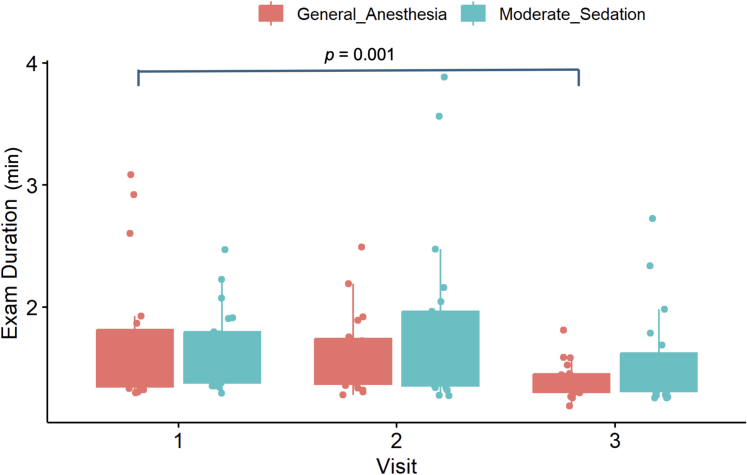
Exam Duration (Minutes) During the Go/No Go Task A box-plot graph panel to demonstrate the performance of the general anesthesia and moderate sedation groups on the Go/No Go task as measured by exam duration (minutes).

Processing speed on the Go/No Go task was assessed by the total exam duration (minutes). The GA group showed a mild statistically significant improvement long-term (V1 vs V3: 1.4 [1.3-1.8] vs 1.4 [1.3-1.5], *P* = 0.001). The MS group also improved over time, although it did not reach statistical significance (V1 vs V3: 1.6 [1.4-1.8] vs 1.4 [1.3-1.6], *P* = 0.100). No short-term (V1 vs V2) differences were found within groups performance (*P* > 0.050) (Figure 10).

## Discussion

To our knowledge, this is the first study to explore the cognitive benefits of avoiding GA in LAAO patients. Our findings indicate improvement in memory-based oculomotor metrics postprocedure occurred more frequently in patients treated under MS compared to those under GA. This proof-of-concept study underscores the significant impact of sedation techniques on cognitive outcomes in elderly LAAO patients (Central Illustration).

**Central Illustration:**
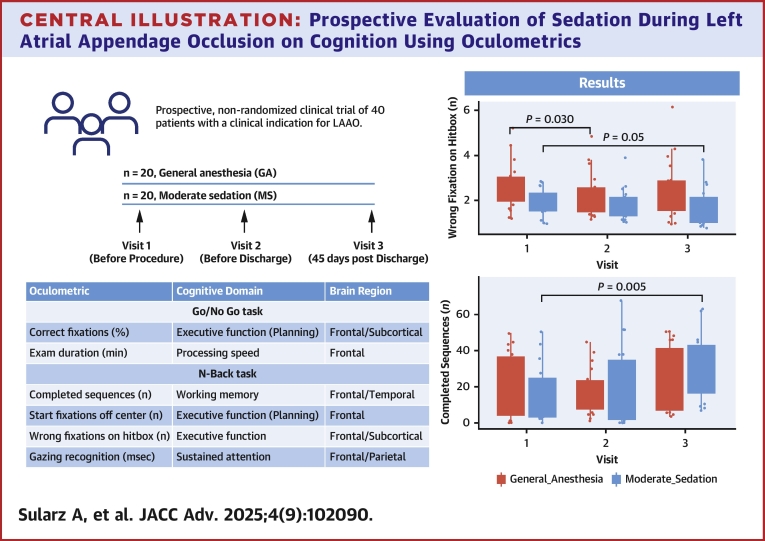
Prospective Evaluation of Sedation During Left Atrial Appendage Occlusion on Cognition Using Oculometrics Prospective evaluation of the impact of sedation during left atrial appendage occlusion on cognitive function using oculomotor biomarkers. We assessed cognitive performance following left atrial appendage occlusion performed under general anesthesia vs moderate sedation, using several digital oculomotor biomarkers. Forty patients (n = 20 general anesthesia, n = 20 moderate sedation) were prospectively enrolled and tested at baseline (V1), 6 hours postprocedure (V2), and 45 days (V3) using Go/No Go and N-Back tasks. Patients in the moderate sedation group showed significant long-term (V1 vs V3) improvement in working memory (Δ *n* complete sequences, *P* = 0.005) and processing speed (Δ *n* wrong fixations V1 vs V3, *P* = 0.050), as measured by the N-Back task while there were no differences in the general anesthesia group (*P* = 0.180 for Δ *n* complete sequences, *P* = 0.410 for Δ *n* wrong fixations on hitbox, and *P* = 0.310). This proof-of-concept study demonstrates the utility of oculomotor-based biomarkers in assessing cognition periprocedurally. LAAO = left atrial appendage occlusion.

The choice between GA vs MS strategy is a subject of an ongoing debate in LAAO research. Our group has previously evaluated the benefits of same-day discharge, reduced hospital stays, and healthcare costs.^4^^,^^21^ MS has been associated with reduced total lab time, enhanced procedural efficiency, and iodine contrast preservation.^3^^,^^22^ However, to date, the impact of sedation strategy on cognitive outcomes following LAAO remains largely unexplored, with most insights drawn from transcatheter aortic valve replacement (TAVR) studies. For example, Mayr et al^23^ found no significant differences in neurocognitive outcomes between MS and GA for TAVR, using 3 visual question-based neurocognitive tests conducted preprocedure and on the seventh postoperative day. These results were later replicated in the SOLVE-TAVI trial,^24^ which utilized a similar battery of tests.

Despite both studies showing no differences in “hard” outcomes such as mortality and morbidity between sedation types, there has been limited focus on “softer” outcomes, such as postoperative cognitive deficits and delirium.^25^

To date, assessing the cognitive impact of sedation regimens after angiographic procedures has been challenging due to the lack of sensitive evaluation tools. Jurga et al^26^ reported no significant cognitive impairment following coronary angiography or percutaneous coronary intervention based on Montreal Cognitive Assessment (MoCA) testing. In contrast, Scott et al^27^ found that left heart catheterization was associated with cognitive decline in 8% to 13% of patients 3 months postprocedure, with baseline cognitive impairment and diabetes mellitus emerging as significant predictors of poor outcomes. A meta-analysis by Ghezzi et al^28^ of cognitive outcomes in TAVR patients yielded similar findings. A key limitation of prior studies is their reliance on MoCA, which, while sensitive to mild cognitive impairment, may miss subtler changes.^29^^,^^30^ Although our nonrandomized design prevented direct group comparisons, several patterns emerged. Notably, the MS group showed significant improvements in working memory and executive function (N-Back task) between V1 and V3, suggesting long-term cognitive benefits. While some learning effect is expected in cognitive testing, the GA group showed no significant performance gains, indicating impaired learning performance. Furthermore, we noted comparable trends in executive performance across both tasks, which supports internal validity of the metrics selected in this study.

Our finding of mild improvement in processing speed on the Go/No Go task for the GA group requires a more nuanced explanation. As such, both the GA and MS groups showed a long-term improvement, although in the MS group the results did not reach statistical significance. This may be due to a small sample size and high variability at baseline. Alternatively, Go/No Go task may be less sensitive than N-Back in distinguishing between the effects of sedation type. Further analysis of the cortical regions involved in each test (Table 1) should provide insight into the mechanism of the pattern of cognitive performance reported here. The effects of different sedative agents on specific brain regions are beyond the scope of this discussion.

Our findings have important potential practical implications. Identifying cognitive markers that can be easily implemented in clinical perioperative settings, such as with a portable headset, could directly enhance patient care. A portable sensitive neurocognitive tool has promising applications in personalized medicine, enabling anesthesia to be tailored to individual risk profiles to minimize sedation burden and mitigate cognitive decline. To validate these findings and establish whether MS is truly superior to GA during LAAO, a prospective, randomized controlled trial is essential. Nevertheless, these findings may have broader relevance to other areas of structural heart disease and electrophysiology, including emerging therapies for atrial fibrillation such as pulsed field ablation.

### Study Limitations

A key limitation of this pilot study is the relatively small sample size, which affects its generalizability. Additionally, the study design involved a relatively short follow-up period, with cognitive function assessed only up to 45 days postoperation. This timeframe may be inadequate to assess long-term cognitive effects, especially in elderly patients. Furthermore, the study did not account for potential confounding factors such as comorbidities, medication use, or baseline cognitive differences, which are known to influence cognitive outcomes. While learning effects over time may account for some improvement in cognitive task performance, they would be expected to affect both groups similarly—unlike the differential pattern observed in this study. Another significant limitation is the lack of randomization between the groups, which precluded a direct comparison of cognitive outcomes. However, the comparison within the same subject (V1, V2, V3) should have minimized this issue. Furthermore, we did not include standardized cognitive screening tools such as MoCA. However, as a pilot study, our primary objective was to assess the feasibility of a novel oculomotor-based approach targeting specific cognitive domains—such as working memory and processing speed—which typically are not captured by these conventional assessments.

## Conclusions

Memory-based oculomotor metrics improved postprocedure in LAAO patients under MS, while no changes were observed in the GA group. This proof-of-concept study highlights the differential impact of sedation strategies on cognitive function in elderly LAAO patients.

## Funding support and author disclosures

Drs Verge, Shulz, and Fernandez are employed by ViewMind. All other authors have reported that they have no relationships relevant to the contents of this paper to disclose.

Perspectives **COMPETENCY IN PATIENT CARE AND PROCEDURAL SKILLS:** Moderate sedation during left atrial appendage occlusion was associated with significant improvement in working memory and processing speed, suggesting a potential cognitive benefit over general anesthesia in older adults. Portable oculomotor-based digital tools provide objective domain-specific cognitive measurements that may be more sensitive to short-term procedural effects than traditional screening methods, although this requires further study. Incorporating perioperative cognitive monitoring into structural heart workflows could support individualized anesthesia planning, particularly in high-risk or frail populations.

**TRANSLATIONAL OUTLOOK:** This study demonstrates the feasibility of using digital oculomotor biomarkers to capture cognitive changes in the peri-operative period following left atrial appendage occlusion. Validation studies should compare oculomotor metrics with standard neurocognitive assessments to establish their clinical relevance and sensitivity. Randomized trials are needed to determine whether sedation strategy causally influences cognitive outcomes and to define which cognitive domains are most affected.
